# The mtDNA haplogroup P of modern Asian cattle: A genetic legacy of Asian aurochs?

**DOI:** 10.1371/journal.pone.0190937

**Published:** 2018-01-05

**Authors:** Aoi Noda, Riku Yonesaka, Shinji Sasazaki, Hideyuki Mannen

**Affiliations:** Laboratory of Animal Breeding and Genetics, Graduate School of Agricultural Science, Kobe University, Kobe, Japan; Universita degli Studi di Pavia, ITALY

## Abstract

**Background:**

Aurochs (*Bos primigenius*) were distributed throughout large parts of Eurasia and Northern Africa during the late Pleistocene and the early Holocene, and all modern cattle are derived from the aurochs. Although the mtDNA haplogroups of most modern cattle belong to haplogroups T and I, several additional haplogroups (P, Q, R, C and E) have been identified in modern cattle and aurochs. Haplogroup P was the most common haplogroup in European aurochs, but so far, it has been identified in only three of >3,000 submitted haplotypes of modern Asian cattle.

**Methodology:**

We sequenced the complete mtDNA D-loop region of 181 Japanese Shorthorn cattle and analyzed these together with representative bovine mtDNA sequences. The haplotype P of Japanese Shorthorn cattle was analyzed along with that of 36 previously published European aurochs and three modern Asian cattle sequences using the hypervariable 410 bp of the D-loop region.

**Conclusions:**

We detected the mtDNA haplogroup P in Japanese Shorthorn cattle with an extremely high frequency (83/181). Phylogenetic networks revealed two main clusters, designated as Pa for haplogroup P in European aurochs and Pc in modern Asian cattle. We also report the genetic diversity of haplogroup P compared with the sequences of extinct aurochs. No shared haplotypes are observed between the European aurochs and the modern Asian cattle. This finding suggests the possibility of local and secondary introgression events of haplogroup P in northeast Asian cattle, and will contribute to a better understanding of its origin and genetic diversity.

## Introduction

All modern cattle are derived from the wild ancestral aurochs (*Bos primigenius*). The aurochs were distributed throughout large parts of Eurasia and Northern Africa during the late Pleistocene and the early Holocene and went extinct in 1627 because of overhunting and habitat contraction [1,2]. Modern cattle are categorized into two species: *Bos taurus* and *Bos indicus*. *B*. *taurus* and *B*. *indicus* have been domesticated independently, the first 10,000–11,000 years in the Upper Euphrates Valley, the second about 2,000 years later in the Indus Valley [3–8].

The bovine mtDNA sequences revealed well-diverged, major haplogroups T and I in *B*. *taurus* and *B*. *indicus*, respectively. Using the mtDNA D-loop sequences, *B*. *taurus* mtDNA was categorized into five sub-haplogroups (T, T1, T2, T3 and T4) and *B*. *indicus* mtDNA into two sub-haplogroups (I1 and I2) [8,9]. The frequency and geographic distributions of the T lineages suggested a single ancestral population source in the Near East and a later spread of *B*. *taurus* [7]. In more recent years, sequences of whole mitochondrial genome (mitogenome) indicated these sub-haplogroups as a single macro-haplogroup T, comprising two clades, T1’2’3’ (also including T4 as sub-clade within T3) and T5 [10,11]. In addition, whole mitogenome analysis also allowed to estimate a predomestic divergence between *B*. *taurus* and *B*. *indicus*, with the divergence time of 330,000 years [12].

In addition to the two major haplogroups T and I, five other haplogroups P, Q, R, C and E have been identified. Haplogroup Q has been detected in ancient domestic cattle [13] and modern cattle in Eurasia and Africa [9,11,12], and it is closely related to haplogroup T [14]. Haplogroup R has only been observed in modern Italian cattle and is phylogenetically distinct from haplogroups P, Q, and T [14]. Haplogroup C was reported in ancient northeast Chinese cattle dated to 10,660 BP [15]. Haplogroup E was identified in an aurochs (<6,000 BP) from Germany [16].

Haplogroup P was one of the major haplogroups in European aurochs but has not been detected in modern cattle in Europe. However, haplogroup P has only been identified in three modern Asian cattle, two Korean cattle and one Chinese cattle [9,17] using a dataset in excess of 3,000 haplotypes [17]. Therefore, haplogroup P in modern cattle is considered to be a remnant of introgression from wild aurochs into the early domesticated cattle gene pool [9,15]. The identification and analysis of these non-T haplogroups could be useful tools for evaluating independent cattle domestication events and/or additional gene introgression from wild aurochs other than those in the Fertile Crescent and the Indus Valley, such as those described in a previous study on the origin of haplogroup R [14].

Here, we report mtDNA diversity of the modern Japanese Shorthorn, which is one of the Japanese Wagyu breeds now bred in the northern part of Japan, by using complete D-loop sequence. Mitogenome sequence has substantial amount of genetic information to reveal the fine phylogenetic structure and estimate more precise haplogroup coalescence times [10,11,12], while the analysis by D-loop sequence has still efficient research advantage in terms of first large-scale survey for unknown population. As a result, Japanese Shorthorn has mtDNA haplogroup P with a surprisingly high frequency. We also report the genetic diversity of haplogroup P compared with the sequences of extinct aurochs. Since haplogroup P has been rarely observed in modern cattle, its unexpected discovery in Japanese Shorthorn represents an opportunity to evaluate the haplogroup genetic diversity and make first-hand hypotheses on its origin.

## Results

We have sequenced the complete D-loop sequences of 181 Japanese Shorthorn (Accession numbers: LC314237-LC314271) and analyzed these in conjunction with previously published mtDNA sequences deposited in DNA Data Bank of Japan (DDBJ) database. S1 Fig indicates the alignment of the D-loop sequences with the bovine reference sequence (BRS) (Accession number V00654). In total, we observed 54 variants, including five transversions, 46 transitions, and three indels. On the basis of these variants, Japanese Shorthorn showed 35 mitochondrial haplotypes.

**Fig 1:**
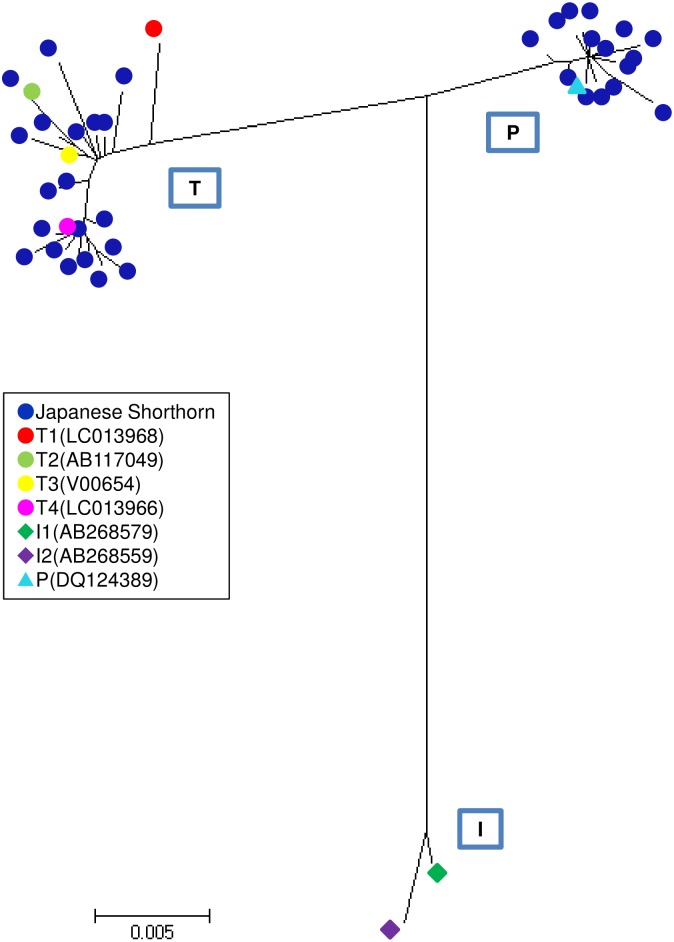
The neighbor-joining tree of Japanese Shorthorn mtDNA complete D-loop sequences. The tree was constructed with representative sequences of the mtDNA haplogroups (T1–T4, I1–2 and P).

Fig 1 shows a phylogenetic reconstruction of Japanese Shorthorn with representative sequences of mtDNA haplogroups (T1–T4, I1, I2 and P). As a result, 54.1% (98/181) of Japanese Shorthorn mtDNAs belonged to *B*. *taurus* mtDNA sub-haplogroups T2 (2/181), T3 (12/181) and T4 (84/181) as previously defined [7,18,19]. Interestingly, 45.9% (83/181) of them belonged to haplogroup P. Haplogroup P is generally found in ancient DNA samples from European wild aurochs remains [20,21], and also observed in only three modern individuals from China and Korea [9], in spite of a survey from thousands of individuals in databases. It was very surprising that the rare haplogroup P in modern cattle was observed in Japanese Shorthorn with an extremely high frequency. In Japanese Shorthorn, 15 haplotypes were observed: JSH21 was predominant and represented 35 times, JSH22 was second most abundant with 11 occurrences, followed by JSH23 and JSH24 with seven (S1 Fig).

**Fig 2:**
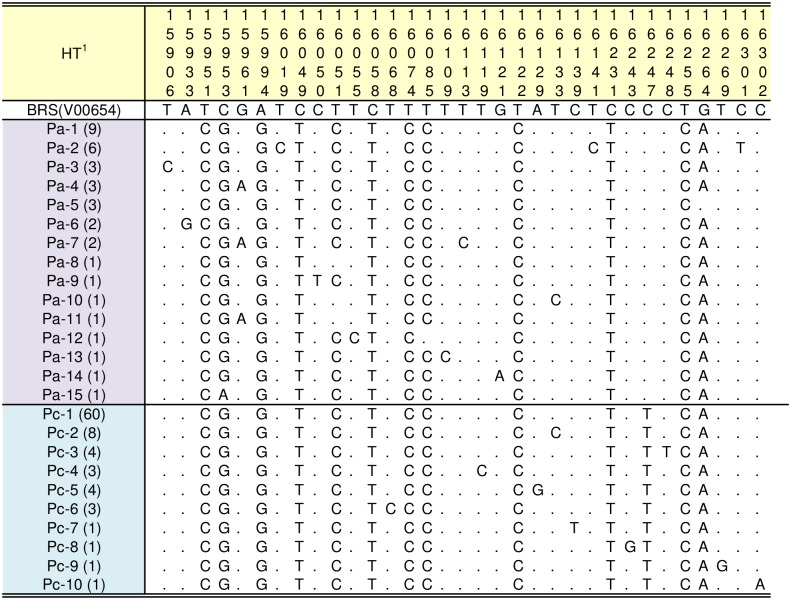
Sequence variation observed among 122 *Bos* samples with haplogroup P. The samples were from 86 modern cattle (83 Japanese Shorthorn, two Korean cattle, and one Chinese Holstein) and 36 archaeological aurochs. They were aligned with BRS (V00654) using the hypervariable 410 bp (bp 15903–16313). Sequence codes and numbers are given in the first column. Only variable sites, with sequence positions given above, are shown. Identity with the first sequence is denoted by a dash substitution by a different base letter. The haplogroup P of European aurochs (Pa) and modern Asian cattle (Pc) were defined by the network analysis shown in Fig 3. 1HT: haplotype using the hypervariable 410 bp sequence.

Subsequently, we compared these P haplotypes of Japanese Shorthorn with those of 36 previously published European aurochs and the three modern Asian cattle sequences [7,9,16] using the hypervariable 410 bp (bp 15,903–16,313) of the D-loop region (Fig 2). The locations and estimated dates of the samples are summarized in S1 Table.

**Fig 3:**
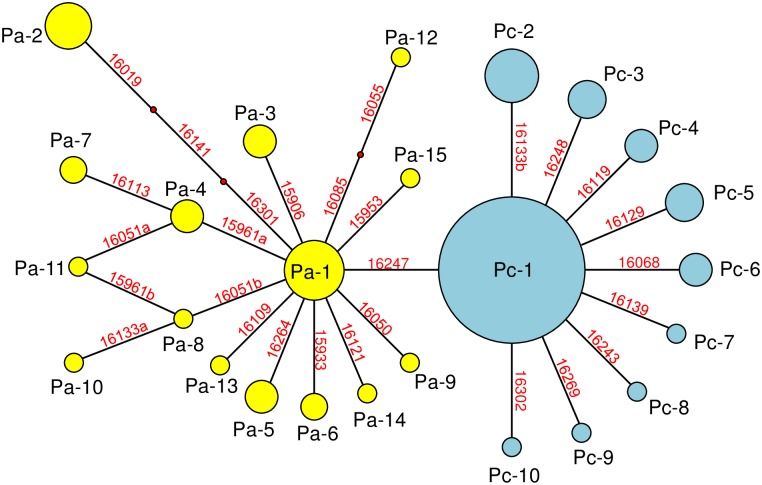
A reduced median network of mtDNA haplogroup P using the hypervariable 410 bp. The network was constructed from 122 *Bos* samples (86 modern cattle samples: 83 Japanese Shorthorn, two Korean cattle, and one Chinese Holstein, and 36 archaeological aurochs samples). The number of times each variant is represented is proportional to the area of its circle. Lines connecting sequence nodes denote substitutions. Numbers with red color indicate substitution positions. Small red circles represent hypothetical sequences which have not been found in the sequencing exercise.

Fig 3 is a reduced median network of mtDNA haplogroup P with D-loop hypervariable 410bp sequences of modern cattle and ancient aurochs. Two main clusters are observed at the base of the branching points for modern Asian cattle and European aurochs at bp 16,247. Here we designated the clusters comprising P aurochs from Europe as cluster Pa and the one with modern Asian P cattle as cluster Pc. No shared haplotypes are observed between the European aurochs (Pa) and the modern Asian cattle (Pc).

The major feature of modern Asian cluster Pc was a marked starlike appearance with 9 haplotypes stemming out of a single predominant haplotype (Pc-1) represented by 60 individuals (0.72). Noticeably the 9 haplotypes were all separated from Pc-1 by single mutations. The other nine haplotypes spread concentrically around the Pc-1 with one substitution. Haplogroup P in modern Korean and Chinese cattle also belonged to modern Asian cluster Pc. A modern Korean (DQ124389) and Chinese (AY998840) individual showed Pc-1 and another Korean individual (AY337527) showed Pc-10 haplotypes.

The European aurochs cluster Pa also formed a starlike tree centered around a main haplotype (Pa-1), and showed a more complex pattern, with a network of intermingled cattle specimens from several sampling locations (Britain, France, Germany, Slovenia, Slovakia, Hungary, Austria and Poland) and of differing ages (dated from 100 BC to 12,000 BP; see details in S1 Table). Therefore, geographical and time differentiations were not detected [16].

S2 Fig is also a network tree using complete D-loop sequences of Japanese Shorthorn haplogroup P. This figure indicates the more variable sequences of haplogroup Pc, with a maximum of five substitutions among the haplotypes (e.g., JSH34 and JSH29).

## Discussion

In the present study, we analyzed mtDNA sequences of 181 Japanese Shorthorn. In half of the Japanese Shorthorn, we confirmed a similar mtDNA topology with other Japanese cattle breeds (Japanese Black, Japanese Brown and Japanese Polled) [18,19,22,23], consisting of sub-haplogroups of T2, T3 and predominantly T4. In addition, unexpectedly, we detected mtDNA haplogroup P in Japanese Shorthorn with an extremely high frequency (83/181). Haplogroup P is generally the most common haplogroup in extinct European aurochs and has not been detected in modern European cattle yet, while the haplogroup P has previously been identified in only three modern Asian cattle individuals from Korea and China [9,17].

### Phylogenetic topology of haplogroup P in modern cattle and aurochs

Modern Asian cluster Pc (Fig 3) including 83 Japanese Shorthorn, 2 Korean cattle and 1 Chinese Holstein formed a starlike pattern with a center of Pc-1 (60 individuals). The starlike topology was similar to those of sub-haplogroups T3 in European and T4 in Japanese cattle [7,18,19], suggesting a past population expansion of haplogroup P as described by a previous study [16]. In addition, shallow genetic diversity (starlike phylogeny and a high proportion identical; see Fig 3 and S2 Fig) among the Pc haplotypes in Japanese Shorthorn was observed. The nucleotide diversity using the complete D-loop sequence of the Pc haplogroup was calculated as 0.0023 in this study, while that of T4 with four Japanese native breeds (n = 179), was calculated as 0.0010 [18,19,22,23], indicating a relatively lower diversity compared to those of Europe (T3) and Africa (T1) (0.0037–0.0043) [18]. The shallow genetic diversity within cluster Pc might be the result of a bottleneck caused by migration to the Japanese Islands, and this may be the same for sub-haplogroup T4 in Japanese breeds.

The cluster of the European aurochs Pa also formed a starlike pattern centered around haplotype Pa-1 and was separated from the Asian cluster Pc by a single substitution at bp 16,247. However, it appeared more complex and structured to cluster Pc and consisted of intermingled samples from several locations and ages of the cattle specimens (S1 Table). This result suggests a more divergent and phylogenetically complex wild population [17,24,25]. Clusters Pa and Pc did not intermingle with each other at all. This might suggest that Pa and Pc had disparate geographical origins with different habitats and genetic lineages.

### Genetic background of Japanese Shorthorn

Japanese Shorthorn is one of the Japanese Wagyu breeds and is raised in the Tohoku and Hokkaido regions in the northern part of Japan. Currently, they are maintained in small populations with approximately 8,000 head total. The Japanese Shorthorn has a genetic background derived from crossbreeding with European and American Shorthorn since the late-19th to the mid-20th centuries [26], and the genetic influence has been confirmed by SNP analysis [27]. Since no haplogroup P has been detected in any modern European breeds so far [5,7,18,19,28,29], the Japanese Shorthorn cattle belonging to cluster Pc might represent an Asian haplogroup P derived from ancestors of Japanese native cattle.

In general, it is considered that the ancestors of Japanese cattle migrated from North China via the Korean peninsula to Japan around the 2nd century AD and then expanded from the western region to all of Japan. This cattle movement was accompanied by the introduction of rice cultivation [18]. All mtDNA of the other Japanese Wagyu breeds (Japanese Black, Japanese Brown and Japanese Polled) belong to the common taurine haplogroup T [18,19,22,23]. However, the ancestral native cattle of the Japanese Shorthorn, which is called “Nambu”, is considered to have a different propagation root from the other Japanese native cattle. Two old historical documents (Nambushi Kyuuki and Tohoku Taiheiki), written in the 16^th^ and 17^th^ centuries, describe that hundreds to thousands of cattle and horses were imported from Mongolia and Siberia in 1454–1456 to the northern part of Japan [30]. The cattle migration might have influenced the genetic material of the “Nambu” cattle. The shallow genetic diversity among modern Asian cluster Pc might be a result of a bottleneck effect by the cattle migration to the Japanese Islands. Therefore, it is probable that cluster Pc in Japanese Shorthorn was derived from a different lineage of Japanese native “Nambu” cattle, which had a genetic influence on the cattle descended from the northeast Eurasian continent.

### Origin of haplogroup P in modern Asian cattle

Here, we identified the haplogroup P in Japanese Shorthorn with an extremely high frequency. To date, haplogroup P in modern cattle has been explained by rare introgression events between female European aurochs and domesticated cattle from the Near East [12,15]. However, our finding may give additional interpretations on where haplogroup P in modern Asian cattle originated. It is unlikely that genetic diversity with haplogroup P originates from the initial domestication events [17]. Thus, the introgression of P might have occurred somewhere on the trajectory of cattle from the Near East to East Asia. Moreover, northeast Chinese cattle specimens, dated from before 10,000 years ago, provide evidence that humans were managing local taurine cattle, which had the genetically distinct and unique mtDNA haplogroup C [15]. This evidence suggested the possibility of local and secondary introgression events in northeast Asia from aurochs. Therefore, the Asian haplogroup P may originate from the ancient Asian population of aurochs.

Previous reports have shown that Siberian native “Yakutian” cattle are of T haplogroups and not P like Mongolian native cattle [19,31,32]. Ancestors of Yakutian cattle are considered to trace back to indigenous cattle in Siberia, which migrated 1,000 years ago from the southern Baikal region to the northern regions. However, mtDNA information in northeast Eurasian cattle is still limited. In order to elucidate the origins of haplogroup P in modern Asian cattle, a large-scale survey of northeast Eurasian cattle, e.g. Siberian and northern Mongolian cattle, is required.

To date, whole mitogenome sequences of haplogroup P were obtained only in three samples (two aurochs and one modern cattle) [9,20,33] due to degraded aurochs specimens and rarely observed modern cattle. Therefore, analysis of complete mitogenome for 15 haplotypes of P detected in present study would probably shed light on the actual origin, diversity of the haplogroup P and clarify the relationship between Asian and Japanese P sequences. Finally, Japanese Shorthorn may be an important genetic resource as a breed that includes parts of the ancient genome of aurochs with the haplogroup P.

## Material and methods

### Ethics statement

Ethical approval was generally not required for this study. All blood and nasal samples collections were collected by veterinarians or individual livestock owners. The samples used in this study were collected specifically and solely for the purposes of this study. These treatments were carried out in accordance with Japanese Veterinarians Act (Act No. 186 of 1949).

### Animals

We used 181 DNA samples of Japanese Shorthorn selected randomly from Iwate (134 animals) and Aomori prefectures (47). DNA was extracted from either blood or nasal samples.

### Sequencing

We amplified the complete D-loop region of mtDNA using primers constructed from the cytochrome b (5′- ACAACTAACCTCCCTAAGACTC-3′) and 12S rRNA (5′- GATTATAGAACAGGCTCCTC-3′) gene sequences. The mtDNA amplification and sequencing were carried out according to previous studies [19,34]. Variations in the D-loop region of Japanese Shorthorn were defined by comparison with BRS (accession number V00654) [35].

### Sequence analysis

Sequence alignment of the D-loop region was achieved using the MEGA package Ver. 6.06 [36]. To investigate the genetic relationship among mitochondrial sequences, un-rooted neighbor-joining phylogenetic tree [37] was constructed using the Tamura-Nei distance [38]. The distance computation and phylogenetic tree construction are incorporated in MEGA package Ver. 6.06 [36]. Sites containing alignment gaps were excluded from the analysis. Reduced median networks were constructed by NETWORK 5.0 [39].

For constructing phylogenetic trees, we used representative sequences of mtDNA haplotypes T1 (LC013968), T2 (AB117049), T3 (V00654), T4 (LC013966), P (two European aurochs: JQ437479 and GU985279; two Korean cattle: DQ124389 and AY337527; and one Chinese Holstein: AY998840), I1 (Bhutanese native cattle: AB268579) and I2 (AB268559). We also used 36 European aurochs partial sequences previously reported [16] for constructing a reduced median network.

## Supporting information

S1 Fig Sequence variation observed among 181 Japanese Shorthorn using complete D-loop sequences. Complete D-loop sequences (bp 15792–363) of 181 Japanese Shorthorn were aligned with BRS (V00654). Haplogroups in the second column were determined by the unrooted neighbor-joining tree (Fig 1) and representative mutational motifs of bovine D-loop sequences [12]. ^1^HT: haplotype using complete D-loop sequence, ^2^HG: Haplogroup.
(XLS)

S2 Fig A reduced median network of 83 Japanese Shorthorn using complete D-loop sequences. The network was constructed by complete D-loop sequences (bp 15792–363) of 83 Japanese Shorthorn with haplogroup P. The number of times each variant is represented is proportional to the area of its circle. Lines connecting sequence nodes denote substitutions. Numbers with red color indicates substitution positions. Small red circles represent hypothetical sequences, which have not been found in the sequencing exercise. The topology of the tree enforces the splitting of substitutions into separate events, these are denoted by a, b and c.
(TIF)

S1 Table List of mtDNA haplogroup P. These samples are obtained from the following: a) European aurochs [16] and b) modern Asian cattle [this study, 9,17]. Information on archeological location, age, date, and radiometric date were cited from a previous study [16].
(XLS)
