# Supplementary figures and images for: The mtDNA haplogroup P of modern Asian cattle: A genetic legacy of Asian aurochs?
Source: PLoS One. 2018 Jan 5;13(1):e0190937. doi: 10.1371/journal.pone.0190937 (PMC5755918; doi:10.1371/journal.pone.0190937)

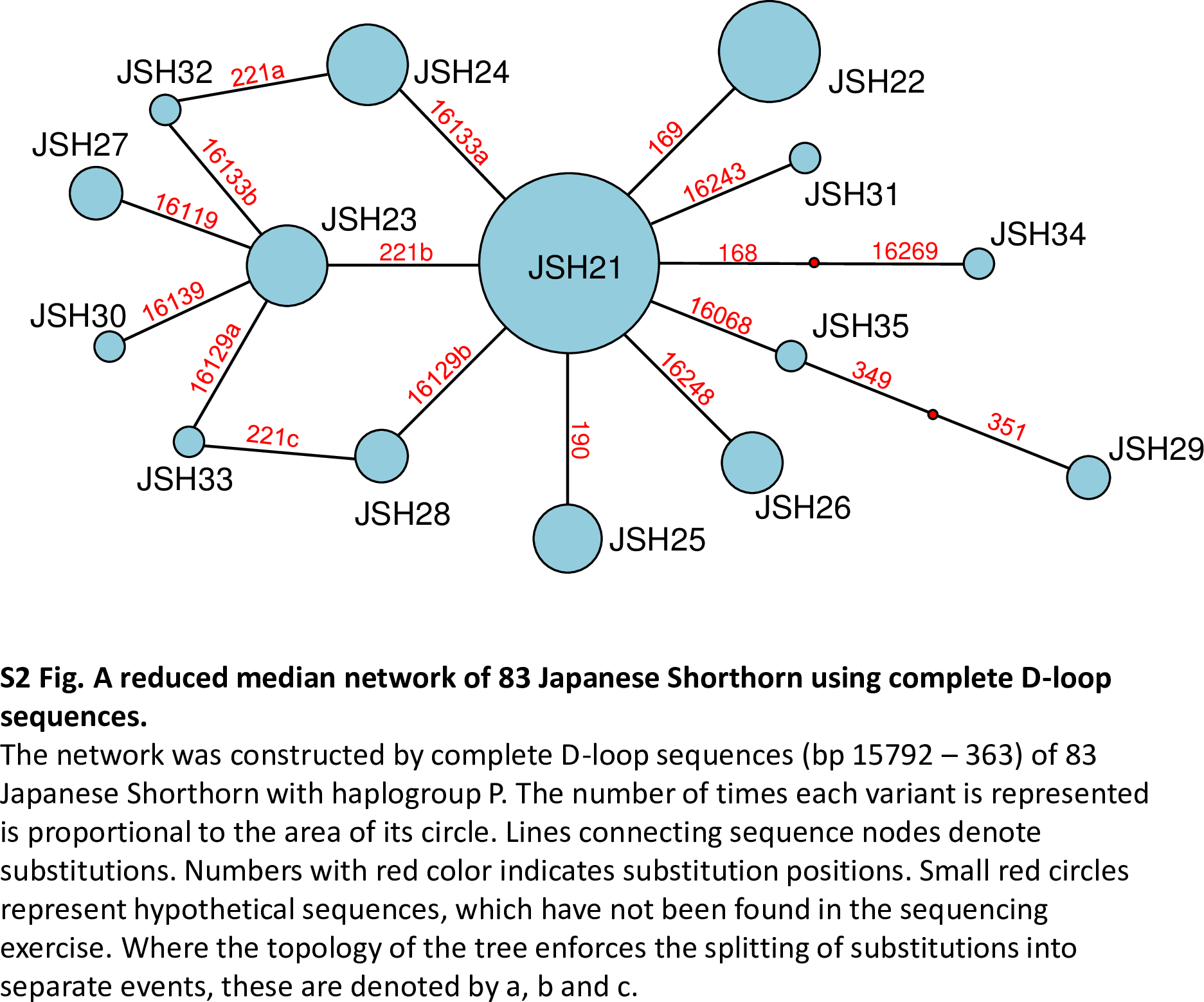

Supplement: S2 Fig — The network was constructed by complete D-loop sequences (bp 15792–363) of 83 Japanese Shorthorn with haplogroup P. The number of times each variant is represented is proportional to the area of its circle. Lines connecting sequence nodes denote substitutions. Numbers with red color indicates substitution positions. Small red circles represent hypothetical sequences, which have not been found in the sequencing exercise. The topology of the tree enforces the splitting of substitutions into separate events, these are denoted by a, b and c. (TIF) [file pone.0190937.s002.tif]
